# Substance use disorder of equimolar oxygen-nitrous oxide mixture in French sickle-cell patients: results of the PHEDRE study

**DOI:** 10.1186/s13023-024-03133-w

**Published:** 2024-03-18

**Authors:** Marie Gérardin, Morgane Rousselet, Marie-Laure Couec, Agathe Masseau, PHEDRE Group, Christelle Chantalat, Françoise Driss, Corinne Guitton, Karima Debbache, Elena Foïs, Frédéric Galacteros, Anoosha Habibi, Anne Léon, Sarah Mattioni, Aline Santin, Scylia Alexis-Fardini, Edima Augusty, Marie Billaud, Lydia Divialle-Doumdo, Maryse Etienne-Julan, Nathalie Lemonne, Marie Petras, Cécile Arnaud, Annie Kamdem, Corinne Pondarré, Amélie Passeron, Christian Rose, Pierre Mornand, Assa Niakaté, Marie-Hélène Odièvre, Cécile Dumesnil, Agnès Lahary, Stéphanie Ngo, Line Haustant-Andry, Romana Ifrim, Gylna Loko, Marie-Laure Couec, Agathe Masseau, Violaine Noël, Claire Pluchart, Shanti Amé, Catherine Paillard, Abdourahim Chamouine, Alain Garou, Marie-Rita Andreu, Jean-François Brasme, Martine Gardembas, Marie-Catherine Receveur, Camille Runel-Belliard, Odile Lejars, Jean-Baptiste Valentin, Corinne Armari, Caroline Makowski, Pierre Cougoul, Anne Besançon, Habib Ghnaya, Vanina Giaccobbi, Aurélie Grados, Estelle Jean, Isabelle Thuret, Marie-Françoise Barrault-Anstett, Mohamed Condé, Suzanne N’dizeye, Laurent Holvoet, Guillaume Gondran, Christophe Piguet, Marie-Pierre Castex, Ana Berceanu, Pauline Simon, Wadih Abouchahla, Anne Lambilliotte, Giovanna Cannas, Alexandra Gauthier, Solène Poutrel, Fabrice Monpoux, Pierre Simon Rohrlich, Audrey Barrelet, Jérôme Clouzeau, Adeline Mallard, Valérie Li Thiao Te, Lavinia Merlusca, Etienne Merlin, Marc Ruivard, Damien Bodet, Hyacinthe Johnson, Odile Minckes, Elodie Colomb-Bottollier, Tackwa Khalifeh, Alain Ramassamy, Stanislas Nimubona, Fabienne Toutain, Arnaud Boutet, Julie Graveleau, Samir Harchaoui, Muriel Lalande, Robert Navarro, Aurélie Desbrée, Isabelle Guichard, Liana Carausu, Jean-Richard Eveillard, Julie Machin, Marie-Hélène Pierre, Diane Cerutti, Narcisse Elenga, Aurélie Aquizerate, Nicolas Authier, Sylvie Deheul, Anne Roussin, Joelle Micallef, Samira Djezzar, French Addictovigilance Network (FAN), Nicolas Authier, Alexandra Boucher, Anne-Sylvie Caous, Sylvie Deheul, Amélie Daveluy, Samira Djezzar, Nathalie Fouilhé, Valérie Gibaja, Reynald Le Boisselier, Joëlle Micallef, Stéphanie Pain, Hélène Peyrière, Anne Roussin, Caroline Victorri-Vigneau, Fanny Feuillet, Pascale Jolliet, Marie Grall-Bronnec, Caroline Victorri-Vigneau

**Affiliations:** 1 CHU Nantes, Centre d’Evaluation et d’Information sur la Pharmacodépendance-Addictovigilance (CEIP-A), Service de Pharmacologie Clinique, Nantes Université, 9 Quai Moncousu, 44 093 Nantes Cedex 1, France; 2 CHU Nantes, UIC Psychiatrie et Santé Mentale, Nantes Université, Nantes, France; 3 CHU Nantes, CHU Tours, INSERM, MethodS in Patients Centered Outcomes and HEalth ResEarch, SPHERE, Nantes Université, Tours Université, Nantes, France; 4 CHU Nantes, Service de Pédiatrie et d’Oncologie Pédiatrique, Nantes Université, Nantes, France; 5 CHU Nantes, Service de Médecine Interne, Nantes Université, Nantes, France; 6 PHEDRE investigateurs, PHEDRE protocole de recherche, Nantes, France; 7 CHU Clermont-Ferrand, Service de Pharmacologie Médicale, Clermont Auvergne Université, Clermont-Ferrand, France; 8 CHU Lille, Service de Pharmacologie, Lille Université, Lille, France; 9 CHU Toulouse, Service de Pharmacologie Médicale et Clinique, Toulouse Université, Toulouse, France; 10 APHM, Service de Pharmacologie Clinique, Hôpital de La Timone, Institut de Neurosciences Des Systèmes, Aix-Marseille Université, Marseille, France; 11 APHP Paris, Centre d’Evaluation et d’Information sur la Pharmacodependence-Addictovigilance de Paris, Hôpital Fernand Widal, APHP Paris, Paris, France; 12 French Addictovigilance Network, FAN, Nantes, France; 13 CHU Nantes, DRI, Plateforme de Méthodologie et Biostatistique, Nantes Université, Nantes, France

**Keywords:** Pain, Substance use disorder, Pseudoaddiction, Sickle cell disease, Nitrous oxide

## Abstract

**Background:**

In many countries, nitrous oxide is used in a gas mixture (EMONO) for short-term analgesia. Cases of addiction, with significant misuse, have been reported in hospitalized patients. Patients suffering from sickle cell disease (SCD) could represent a high-risk population for substance use disorder (SUD) due to their significant pain crisis and repeated use of EMONO. The objective of the PHEDRE study was to assess the prevalence of SUD for EMONO in French SCD patients.

**Results:**

A total of 993 patients were included. Among 339 EMONO consumers, only 38 (11%) had a SUD, with very few criteria, corresponding mainly to a mild SUD due to a use higher than expected (in quantity or duration) and relational tensions with the care teams. Almost all patients (99.7%) were looking for an analgesic effect, but 68% of patients were also looking for other effects. The independent risks factors associated with at least one SUD criterion were: the feeling of effects different from the expected therapeutic effects of EMONO, at least one hospitalization for vaso occlusive crisis in the past 12 months and the presence of a SUD for at least one other analgesic drug.

**Conclusions:**

The use of EMONO was not problematic for the majority of patients. Manifestations of SUD that led to tensions with healthcare teams should alert and lead to an evaluation, to distinguish a true addiction from a pseudoaddiction which may be linked to an insufficient analgesic treatment related to an underestimation of pain in SCD patients.

*Trial registration***:** Clinical Trials, NCT02580565. Registered 16 October 2015, https://clinicaltrials.gov/

## Background

### Nitrous oxide: a gas with two sides

Nitrous oxide (N_2_O) has been used in medical practice for many years due to its analgesic and anxiolytic properties [1]. It is also used as a food additive, and it is currently one of the most popular substances used by young people for its euphoric effects. The mechanism of N_2_O is still not fully known [2]; it interacts in particular with opioidergic, GABAergic, dopaminergic and glutamatergic systems. N_2_O is a substance monitored in many countries because of the misuse of cartridges for whipped cream. Dependence on and tolerance to the effects of N_2_O have been demonstrated [3]. Moreover, numerous cases of N_2_O abuse and subsequent adverse effects have been reported in the literature [4–10].

In medical practice, it is used worldwide in a gas mixture with oxygen in the indication of short-term analgesia for painful procedures or for mild to moderate pain in adults and children. An equimolar mixture of oxygen and nitrous oxide (EMONO) has been a marketing authority in France since 2001. Because of the risk of abuse/dependence, it is subject to a risk management plan (RMP) monitored by the national French Addictovigilance Network (FAN) under the responsibility of the Nantes Addictovigilance Centre. Reports of the RMP highlighted cases of addiction or adverse effects following very extensive and prolonged use of the gas [11]. These cases involved patients in a serious medical situation, requiring repeated use of EMONO.

### SCD patients: a vulnerable population

SCD is an inherited red blood cell disorder caused by the presence of abnormal haemoglobin, called haemoglobin S (HbS), which often leads to complications, including vaso-occlusion, that result in tissue ischaemia causing acute, severe pain episodes [12, 13]. In cases of hospitalization for vaso-occlusive crisis (VOC), EMONO is used for the rapid management of pain in combination with morphine in children and adolescents [14, 15] Among SCD patients, pain is subject to significant interindividual and intraindividual variability with respect to the severity, site and duration of the pain episode, which leads to difficulties in management among patients and health care professionals [14, 16]. However, if the profile of SCD patients (young people, recurrent pain, psychiatric comorbidities, exposure to analgesic and psychotropic drugs, etc.) can represent a risk factor for addiction, poor pain management can expose patients to the risk of pseudoaddiction. Addiction is characterized by craving for drug or positive reward, dysfunctional emotional response, failure to recognize significant problems affecting behaviour and relationships, inability to consistently abstain, and impairment in control of behaviour [17]. Pseudoaddiction (defined as a constant fear of being in pain, hypervigilance, usually resolving with pain resolution) [17] occurs when patients attempt to obtain a higher analgesic dose to relieve pain. This behaviour could be interpreted by medical staff as being compatible with substance use disorders (SUDs), but in pseudoaddiction, analgesic-seeking behaviour stops when the pain is treated effectively and does not persist after pain relief has been achieved [18]. The major risk is that these patients receive fewer analgesics; therefore, treatment is not optimal [19–21].

### SCD and nitrous oxide: Addiction or pseudoaddiction?

Currently, there are few data available on this concern, and no studies have been carried out in patients with SCD. Regrettably, potential SUD with EMONO is an issue for patient treatment strategies, and the need for specific evaluation is supported by physicians working on SCD.

The PHEDRE study (Pharmacodépendance et DREpanocytose—drug dependence and SCD) is the first national study aimed at evaluating the use of EMONO in patients with SCD.

## Methods

### Study design

The PHEDRE study protocol was previously described in a publication, freely available online [22]. It was a national, observational, and transversal study based on an interview carried out by the Nantes Addictovigilance Centre and funded by a Grant from the French National Agency for Medicines and Health Products Safety (Agence Nationale de Sécurité du Médicament et des produits de santé – ANSM). It was monitored by a pluridisciplinary steering committee (pharmacologists, psychiatrists specializing in addiction, biostatisticians, and physicians specializing in SCD). This study was approved by the local health ethics committee, the CCTIRS (Comité Consultatif sur le Traitement de l'Information en matière de Recherche dans le domaine de la Santé) and the CNIL (Commission Nationale de l'Informatique et des Libertés). The study is registered as NCT02580565. All participants provided written informed consent (and legal representation for patients under 18 years old) in accordance with the Declaration of Helsinki.

### Patients

In France, patients with SCD can benefit from a follow-up in a Reference Centre or Special Centre for Children and Adults (RSCCA). To be eligible for the PHEDRE study, patients had to (1) have a SCD diagnosis confirmed by genetic testing, (2) be treated in an RSCCA, and (3) have given informed written consent.

Patients under guardianship (under tutelage or curatorship) and those without the general aptitude to participate in the study assessment (*i.e.,* not able to respond to the telephone interview) were not included in the study.

### Study procedures

The patients were recruited by their physician when they came to the RSCCA for their routine follow-up between September 2015 and December 2017. The physician completed a form with the patient’s medical data. Thereafter, patients were interviewed by phone by a trained interviewer. An ad hoc standardized heteroquestionnaire for phone interview was validated by the steering committee. Before starting phone interviews, the interviewer had a specific formation on SCD and pharmacodependence with members of the steering committee and received a notice with instructions on how to ask the questions according to the age of the interviewee and the substance used.

Data from the physician’s form included demographic (sex, age) and clinical data (SCD genotype, hospitalizations for VOC in the past 12 months). Data from the phone interview included socioeconomic data (occupational activity for majors, employment status of mother and father for minors) number of different analgesics used at home in the past 12 months, use of psychoactive medicines or drugs (other than analgesics) in the past 12 months (only for patients age 12 and over), effects sought and felt with EMONO, use of EMONO out of a painful context (i.e., in the hospital but at a time when there was little or no pain) and assessment of SUD for EMONO and other analgesic drugs according to DSM 5 criteria [23].

To assess the effects of EMONO, felt or sought, patients were asked to answer “yes” or “no” to the following proposals: analgesia, sedation, anxiolysis, reduction of negative affects, gliding, well-being and other effects (specify). For the analysis, effects were grouped into categories as follows: “Therapeutic only” when the analgesic effect was the only or when it was associated with sedation and/or anxiolysis but with no other effect; “Gliding” when “high” effects were mentioned regardless of other effects; “Others” for patients who did not fit into either of the above two categories.

**Fig. 1:**
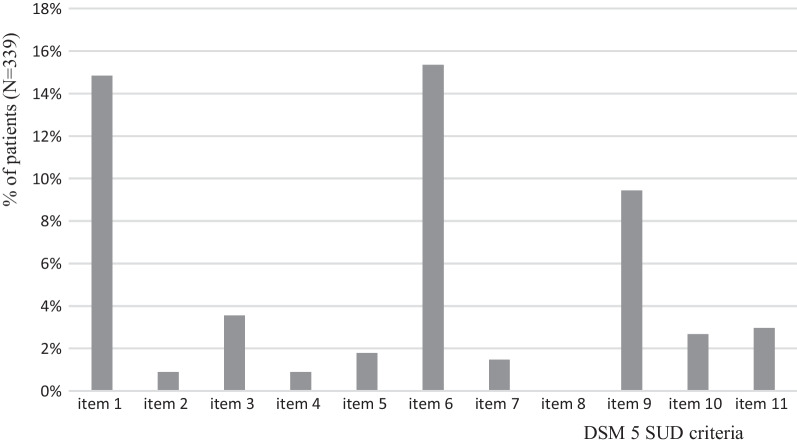
SUD criteria positivity rate. *if there are missing data for some items, the number of patients concerned is mentioned below. DSM SUD criteria: Item 1 = Using more of a substance than planned, or using a substance for a longer interval than desired (missing data for 2 patients). Item 2 = Inability to cut down despite desire to do so (missing data for 2 patients). Item 3 = Spending substantial amount of the day obtaining, using, or recovering from substance use (missing data for 1 patient). Item 4 = Cravings or intense urges to use (missing data for 1 patient). Item 5 = Recurrent substance use may result in a failure to fulfill major role obligations at work, school, or home (missing data for 1 patient). Item 6 = Persistent usage despite persistent or recurrent social or interpersonal problems. Item 7 = Giving up or cutting back on important social, professional, or leisure activities because of use. Item 8 = Using in physically hazardous situations, or usage causing physical or mental harm. Item 9 = Persistent use despite the user's awareness that the substance is causing or at least worsening a physical or mental problem. Item 10 = Tolerance: needing to use increasing amounts of a substance to obtain its desired effects (missing data for 2 patients). Item 11 = Withdrawal: characteristic group of physical effects or symptoms that emerge as amount of substance in the body decreases (missing data for 2 patients) (N=339 patients*)

SUD was assessed using DSM 5 criteria [23] in relation to the context(s) of use: *1)* when the drug was used for analgesic effects in a painful context and *2)* when (if) the drug was used outside a painful context. According to the DSM 5, SUD was defined by meeting at least 2 of the 11 diagnostic criteria. Figure 1 (SUD criteria positivity rate) details SUD criteria. As a general estimate of severity, a “mild” SUD is suggested by the presence of two to three criteria, “moderate” by four to five criteria, and “severe” by six or more criteria.

### Outcomes

The primary outcome was the prevalence of SUD for EMONO according to DSM 5 criteria in a painful context and out of a painful context (if applicable). The secondary outcomes addressed in this publication were the characterization of patients with one or more SUD criteria for EMONO and the identification of factors associated with the presence of these criteria. Indeed, even if only one criterion is present, it is important to identify patients at risk of addiction as early as possible. Other secondary objectives (assessment of prevalence and characterization of SUD for other analgesics) will be the subject of a separate publication.

### Sample size

The aim was to include all patients who met the inclusion criteria in RSCCA who agreed to participate in the study. The study was proposed to the physicians of 79 RSCCA spread over the French territory (72 main centres and 7 annex centres). The total number of patients who could be included in France was thus estimated to be approximately 2000 people.

### Statistical analysis

Continuous data are expressed as the mean (± SD), and categorical data are expressed as numbers and percentages. The primary outcome is expressed as a prevalence, including a confidence interval of 95% (95% CI). Patients with missing data that did not enable SUD evaluation were excluded from the analysis. Univariate analysis was conducted to determine the potential factors associated with the presence of at least one SUD criterion using the chi-square test or Student’s t test as appropriate. Variables identified by univariate analysis with a cut-off at 0.20 were included in a multivariate logistic regression model, and backwards selection was applied. A Hosmer–Lemeshow test was used to test the calibration of the model. The final model is presented with the adjusted odds ratio (OR) and 95% CI.

## Results

### Descriptive analysis

Seventy-three RSCCA participated in the PHEDRE study in metropolitan France and overseas departments. A total of 993 patients were included. Data for 121 patients could not be analysed (not reachable, refusal to answer, etc.), and 872 remained for analysis. Among them, 339 patients (39%) had used EMONO in the 12 months preceding the study. Their main characteristics are described in Table 1.

**Table 1:** Potential factors associated with the presence of at least one SUD criterion – univariate analysis

	All patients (N = 339) n (%) or m (SD)	No SUD criterion (N = 211) n (%) or m (SD)	At least one SUD criterion (N = 125) n (%) or m (SD)	*p* value
*Demographic data*				
**Sex**				
N	339	211	125	
Female	192 (56.6%)	110 (52.1%)	81 (64.8%)	0.02
**Age (years)**				
N	339	211	125	
Mean (SD)	20.2 (9.1)	20.1 (9.1)	20.3 (9.1)	0.81
Min–Max	[5.0;57.0]	[5.0;56.0]	[6.0;57.0]	
Under 18	147 (43.4%)	91 (43.1%)	55 (44.0%)	0.88
Over 18	192 (56.6%)	120 (56.9%)	70 (56.0%)	
*SCD clinical data and analgesics’ use*				
**Genotype**				
N	338	210	125	
Homozygous SS	276 (81.7%)	170 (81.0%)	104 (83.2%)	0.09
**Hospitalizations for VOC (past 12 months)**				
N	339	211	125	
None	46 (13.6%)	37 (17.5%)	9 (7.2%)	0.006
1 or 2	158 (46.6%)	101 (47.9%)	55 (44.0%)	
3 or more	135 (39.8%)	73 (34.6%)	61 (48.8%)	
**Number of analgesic treatments at home**				
N	339	211	125	
≤ 2	140 (41.3%)	93 (44.1%)	46 (36.8%)	0.02
3	175 (51.6%)	109 (51.7%)	64 (51.2%)	
≥ 4	24 (7.1%)	9 (4.3%)	15 (12.0%)	
**SUD for at least one analgesic drug (other than EMONO)**				
N	333	209	121	
Yes	212 (63.7%)	119 (56.9%)	90 (74.4%)	0.002
*Use of psychoactive substances other than analgesics*				
**Tobacco use**				
N	261	161	97	
Yes	31 (11.9%)	21 (13.0%)	10 (10.3%)	0.51
**Alcohol use**				
N	261	161	97	
Yes	82 (31.4%)	54 (33.5%)	27 (27.8%)	0.34
**Psychoactive medications (other than analgesics) use**				
N	262	160	99	
Yes	43 (16.4%)	23 (14.4%)	19 (19.2%)	0.31
*Characteristics of EMONO use*				
**Patients who felt analgesic effect**				
N	339	211	125	
Yes	336 (99.1%)	210 (99.5%)	123 (98.4%)	0.56
Felt effects (categories*)				
N	338	211	125	
“Therapeutic only”	38 (11.2%)	33 (15.6%)	4 (3.2%)	0.002
“Gliding”	257 (76.0%)	150 (71.1%)	106 (84.8%)	
“Others”	43 (12.7%)	28 (13.3%)	15 (12.0%)	
**Patients who sought analgesic effect**				
N	339	211	125	
Yes	338 (99.7%)	210 (99.5%)	125 (100.0%)	1.00
**Patients who sought other effects than analgesic**				
N	339	211	125	
Yes	231 (68.1%)	134 (63.5%)	96 (76.8%)	0.01
Sought effects (categories*)				
N	337	210	125	
“Therapeutic only”	129 (38.3%)	90 (42.9%)	38 (30.4%)	0.02
“Gliding”	177 (52.5%)	98 (46.7%)	78 (62.4%)	
“Others”	31 (9.2%)	22 (10.5%)	9 (7.2%)	
**Intake context**				
N	337	210	124	
Not outside a painful context	336 (99.7%)	210 (100.0%)	123 (99.2%)	0.37

*The effects of EMONO were grouped into categories as follows:

“Therapeutic only” when the analgesic effect was the only or when it was associated with sedation and/or anxiolysis but with no other effect;

“Gliding” when “high” effects were mentioned regardless of other effects;

“Others” for patients who did not fit into either of the above two categories

They were mainly female (57%) and had a mean age of 20.2 years. A total of 147 subjects were minors (43%), in most cases parents were employed (82% for father employment, 68% for mother employment). Among the adults, very few were on disability (4.2%), approximatively one third was students, approximatively one third was employed and approximatively one third was both unemployed and not enrolled in school. They were mainly homozygous for the SCD genotype (82%), and most of them (86%) had been hospitalized at least once for VOC in the past 12 months. The use of analgesics at home was common (nearly 60% had used at least 3 different analgesics in the past 12 months), and SUD for analgesics other than EMONO was frequent (64%).

Concerning felt effects of EMONO, 38 patients (11%) experienced effects in the "Therapeutic only" category (analgesia, anxiolysis, sedation), 257 (76%) experienced a “Gliding” effect (plus or minus other effects) and 43 (13%) were classified in the "Others" category (33 citing effects that could be considered positive (well-being: 32 and reduction in negative affects: (1), and 11 undesirable effects (dizziness: 3, headache: 2, nausea/vomiting: 2, hallucinations: 1, excitement: 1, disorientation: 1, panic: 1).

Concerning sought effects of EMONO, almost all patients (99.7%) were looking for an analgesic effect, but 68% of patients were also looking for other effects. 129 (38%) were seeking “Therapeutic effects only”, 177 (53%) were looking for a “Gliding” effect and 30 (9%) were classified in the "Others" category (30 citations of well-being).

**Fig. 2:**
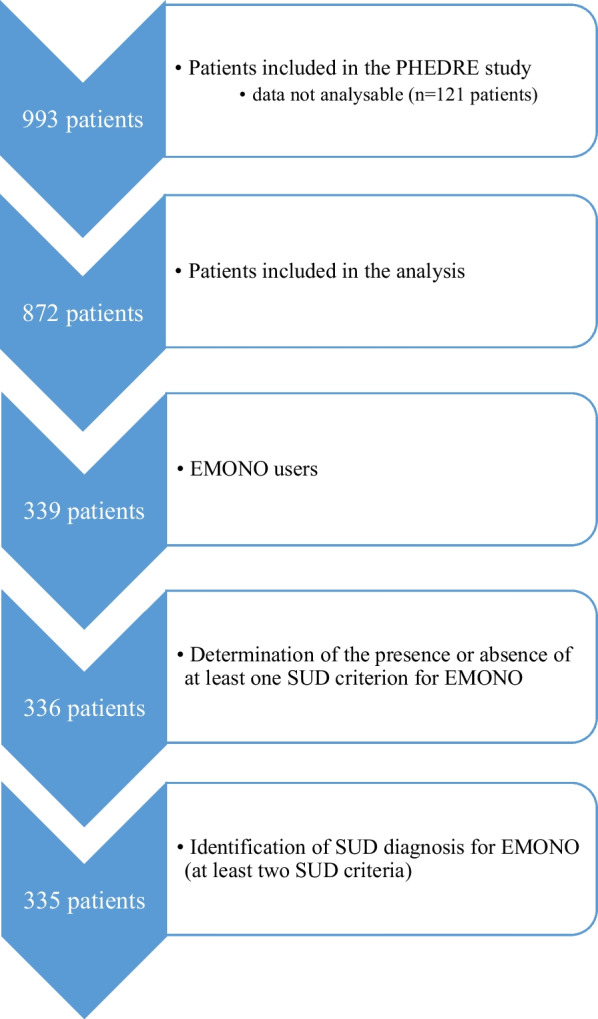
Study population

Due to missing data on SUD criteria, the primary outcome was evaluated in 335 patients (diagnosis of SUD, i.e., at least 2 SUD criteria for EMONO), and the secondary outcome was evaluated in 336 patients (at least one SUD criterion for EMONO). Figure 2 (Study population) details the number of patients included in the analyses.

### Primary outcome: prevalence of SUD for EMONO

A total of 38 patients out of 335 (11.3% CI95% [8.0% to 14.7%]) had a SUD for EMONO (at least 2 criteria). Thirty-three (9.9%) had 2 or 3 criteria, which corresponded to a mild SUD according to DSM 5. Among them, only one patient used EMONO outside of a painful context.

The majority of patients (297; 88.9%) had no SUD for EMONO. Among them, 211 had no criterion for a SUD, and 86 patients (25.8%) had only one.

### Secondary outcomes

#### Characterization of patients with at least one SUD criterion for EMONO

According to Fig. 1, the more prevalent SUD criteria were item 6 (persistent usage despite persistent or recurrent social or interpersonal problems), positive in 15.3% of patients, item 1 (using more of a substance than planned, or using a substance for a longer interval than desired) in 14.8% and item 9 (persistent use despite the user's awareness that the substance is causing or at least worsening a physical or mental problem) in 9.4%.

#### Factors associated with the presence of at least one SUD criterion

The results of univariate comparison between patients with at least one SUD criterion (125; 37.2%) for EMONO and patients with no criteria (211; 62.8%) are presented in Table 1. There was no significant difference in socioeconomic status between patients with at least one SUD criterion and the others (p = 0.19 for occupational activities in adults, p = 0.42 for mother employment in minors, p = 0.28 for father employment in minors).

In the final multivariate logistic regression model (Table 2), the independent risk factors associated with at least one SUD criterion were the feeling of effects different from the “Therapeutic only” (OR = 5.37 for “Gliding” and OR = 3.99 for “Others”), at least one hospitalization for VOC in the past 12 months (OR = 1.87 for “one or two” and OR = 2.79 for “three or more”) and the presence of a SUD for at least one other analgesic drug (OR = 1.97).

**Table 2:** Multivariate logistic regression model—Risk factors for at least one SUD criterion for EMONO (N = 330; Hosmer–Lemeshow p value = 0.8825; AUC = 0.66 [0.61; 0.72])

Variables	Adjusted odds-ratio (OR)	CI_95%_	*p* value
Felt effects (categories*)			0.0083
“Therapeutic only”	Reference	–	–
“Gliding”	5.37	[1.82; 15.83]	
“Others”	3.99	[1.15; 13.80]	
Hospitalizations for VOC (past 12 months)			0.0362
None	Reference	-	
1 or 2	1.87	[0.82; 4.26]	
3 or more	2.79	[1.22; 6.41]	
SUD for at least one analgesic drug other than EMONO	1.97	[1.18; 3.27]	0.0090

*The effects of EMONO were grouped into categories as follows:

“Therapeutic only” when the analgesic effect was the only or when it was associated with sedation and/or anxiolysis but with no other effect;

“Gliding” when “high” effects were mentioned regardless of other effects;

“Others” for patients who did not fit into either of the above two categories

## Discussion

### N_2_O, SCD and addiction: A false issue?

The results of our study showed that approximately 40% of the patients were treated with EMONO, and among them, only 11% had a SUD characterized, in the majority of cases, by only two positive criteria. Recent HAS recommendations [24] call for the safe use of analgesics, particularly opioids, in the management of pain to prevent any risk of misuse and addiction. One of the challenges is to make healthcare professionals aware of the need for early intervention in high-risk situations. In this attitude of early detection, we sought to identify the presence of at least one DSM criterion of SUD among EMONO users and to define the characteristics of the patients concerned (37%).

A higher SUD rate might have been expected. Indeed, SCD patients could be considered presenting addiction risk factors: they are young, in pain and receive recurrent psychotropic and analgesic treatments whose addictive nature is known, but 63% of patients did not present any criteria for SUD for EMONO.

The most frequently positive criteria for SUD were items 1 and 6, namely, “Using more of a substance than planned, or using a substance for a longer interval than desired” and “Persistent usage despite persistent or recurrent social or interpersonal problems”. In the context of EMONO use in hospitals, these criteria revealed situations of tension with medical staff during hospitalizations due to patient requests to obtain EMONO more frequently or to extend the time of administration.

In our study, certain characteristics seemed to be more present among patients presenting at least one SUD criterion for EMONO. First, they felt effects different from therapeutic effects more often, particularly effects that could be considered positive, such as “Gliding”, and therefore could be sought. Then, they were more likely to have severe SCD as they were hospitalized more often. Some authors have shown that SCD patients with extremely high hospital use demonstrated reactive behaviors in seeking care for pain management, which led to a distrustful and dysfunctional patient-provider relationship [25]. Moreover, according to univariate analysis, they used more analgesics at home and were more likely to have SUD for at least one other analgesic drug. No sociodemographic or socioeconomic data collected in the study were associated with presenting at least one SUD criterion for EMONO. Socioeconomic status is known to impact dependence in the general population [26]. It is possible that socioeconomic data collected in PHEDRE study were too limited even if they help to approximate socioeconomic status. To avoid extending the telephone questionnaire excessively and to enhance patient acceptance for the study, we chose to focus on specific variables: the consumption of analgesic medications, data related to patient care, and a few essential sociodemographic data. This decision was made to identify risk factors that we could address, prevent, and make recommendations on, but they are not exhaustive.

### No addiction with an addictive substance

Pharmacologically, N_2_O acts on mediators involved in addictive phenomena, notably dopamine, a mediator of the reward circuit, opioidergic system and GABA, a mediator involved in anxiety. National addictovigilance monitoring has shown potential for abuse and dependence associated with significant daily consumption and harmful consequences, particularly neurological consequences [11]. Patients in the PHEDRE study also reported harmful consequences, as item 9 (persistent use despite the user's awareness that the substance is causing or at least worsening a physical or mental problem) was positive for 9% of patients. However, these consequences corresponded to unpleasant side effects such as "nausea" or "dizziness".

There are several possible explanations for the fact that there were few patients with SUD. First, as our results were based on declarative data in order to get as close as possible to actual patient consumption, but memorization bias and underreporting bias are possible. This risk of bias is one of the limitations of the study. If the patients questioned could not remember the details of their consumption, this was recorded as "missing data". Concerning use outside the context of pain and SUD criteria, it is possible that some respondents did not wish to admit to such misuse.

However, declarative data, despite the risk of bias inherent in this type of data, remains the closest to the reality of consumption by the person surveyed and Addictovigilance centres are used to carrying out studies on the use of psychoactive substances, and to collecting declarative data. To minimize declarative bias, the interviewer was not part of the patient medical team, and the data collected were not communicated to them. This was explained to the patient before starting the questionnaire, along with the fact that his or her answers would have no consequences in terms of therapeutic management. Second, in PHEDRE, it was not about diverted N_2_O but EMONO, a drug subject in France to part of the regulations on narcotics. The low rate of use outside a painful context was predictable because EMONO is not widely available outside the hospital and therefore not easily accessible. This framework and the vigilance of health professionals allow the use to be controlled. However, patients may have requested EMONO during their hospitalization at times when they were in little or no pain, but only one patient was concerned about the use of EMONO at a time when he was not in pain. Above all, patients in the PHEDRE study used EMONO in an acute painful context, which may limit the addictive effects. Therefore, we were unable to compare the prevalence of SUD criteria between intakes in a painful context and those outside a painful context.

### Communicating for better pain management

Situations of tension between caregivers and patients for a probable undertreatment of pain could be related to the lack of knowledge of the characteristics of pain in these particular patients, especially in emergency services. However, no objective measure can assess pain in children and adults with SCD. Classic pain assessments are limited by the momentary assessment of pain and inter individual variability due to differences in pain tolerance [27]. For example, patients who suffer from chronic sickle cell pain may not have the typical physical characteristics of pain and may not appear distressed when performing daily activities [25]. Patients are therefore often confronted with an underestimation of the intensity of their pain by the caregivers. Thus, the rating on a pain intensity scale should never be the sole determinant for the administration of analgesia if it is questioned by the caregiver. The cornerstone of pain management is trust between the affected individual in pain and the health care provider [27]. Where there are sociocultural barriers (e.g., of class, race, ethnicity, language), the communication and assessment of pain can all be more compromised [28].

A better understanding of the concepts of addiction and pseudoaddiction would also help to better understand patients. In the case of repeated requests for analgesics, a precise evaluation must be proposed, including an addictological evaluation of all consumption and, in particular, the place of substances for the subject. Even if patients experience euphoric effects with EMONO and consequently can look for them, our results showed that what motivates the request is the analgesic effect. This is perhaps the main contribution of PHEDRE. The repeated requests for EMONO are also related to the pharmacology of N_2_O, which has a very short-lived effect. Sickle cell pain is said to be worse than postoperative or traumatic pain and is as intense as metastatic bone cancer pain [29, 30]. EMONO is often the first medication administered upon arrival at the hospital to relieve pain, particularly in children [14], inducing a high level of patient expectation for this drug. The memory of previous administrations and the instantaneous effect of EMONO with immediate pain relief, compared to other analgesic therapies with a longer delay of action, could explain the positive effects felt and sought by patients.

## Conclusions

PHEDRE was a large study conducted among SCD patients, that explored SUD for EMONO among these chronic pain patients, which are not comparable to other chronic pain patients. To our knowledge, this exploration has never been carried out elsewhere.

Our results showed that the use of EMONO was not problematic for the majority of patients, which should prompt a change in the modalities of pain management and the reinforcement of psychological care. Manifestations of SUD that led to tensions with healthcare teams should alert and lead to an evaluation to distinguish a true addiction from a pseudoaddiction. If genuine drug addiction to analgesics was identified, it would require treatment adjustment.

## Data Availability

The data that support the findings of this study are available from the corresponding author upon reasonable request. We are urged to be cautious by our Hospital Clinical Research Department regarding the transmission of anonymized data. Moreover, the data collected in this study may include potentially sensitive data (concerning mental health and behavior). Finally, before sending data to other researchers, we must determine that the purpose of the data processing is compatible with the information provided to and consent given by the patients and legal representatives for patients under 18 years old.
